# Pivotal roles of melanopsin containing retinal ganglion cells in pupillary light reflex in photopic conditions

**DOI:** 10.3389/fncel.2025.1547066

**Published:** 2025-02-07

**Authors:** Jeremy Matthew Bohl, Abdul Rhman Hassan, Zachary J. Sharpe, Megi Kola, Angela Shehu, Deborah Langrill Beaudoin, Tomomi Ichinose

**Affiliations:** Department of Ophthalmology, Visual and Anatomical Sciences, Wayne State University School of Medicine, Detroit, MI, United States

**Keywords:** retina, melanopsin ganglion cell, photoreceptor, Cnga3, Gnat1, pupillary light reflex (PLR)

## Abstract

The pupillary light reflex (PLR) is crucial for protecting the retina from excess light. The intrinsically photosensitive retinal ganglion cells (ipRGCs) in the retina are neurons that are critical to generating the PLR, receiving rod/cone photoreceptor signals and directly sensing light through melanopsin. Previous studies have investigated the roles of photoreceptors and ipRGCs in PLR using genetically-modified mouse models. Herein, we acutely ablated photoreceptors using N-nitroso-N-methylurea (MNU) to examine the roles of ipRGCs in the PLR. We conducted PLR and multiple electrode array (MEA) recordings evoked by three levels of light stimuli before and 5 days after MNU intraperitoneal (i.p..) injection using C57BL6/J wildtype (WT) mice. We also conducted these measurements using the rod & cone dysfunctional mice (Gnat1^–/–^ & Cnga3^–/–^:dKO) to compare the results to published studies in which mutant mice were used to show the role of photoreceptors and ipRGCs in PLR. PLR pupil constriction increased as the light stimulus intensified in WT mice. In MNU mice, PLR was not induced by the low light stimulus, suggesting that photoreceptors induced the PLR at this light intensity. By contrast, the high light stimulus fully induced PLR, similar to the response in WT mice. In dKO mice, no PLR was evoked by the low-light stimulus and a slow-onset PLR was evoked by the high-light stimulus, consistent with previous reports. *Ex vivo* MEA recording in the MNU tissue revealed a population of ipRGCs with a fast onset and peak time, suggesting that they drove the fast PLR response. These results suggest that ipRGCs primarily contribute to the PLR at a high light intensity, which does not agree with the previous results shown by mutant mouse models. Our results indicate that the melanopsin response in ipRGCs generate fast and robust PLR when induced by high light.

## Introduction

The pupillary light reflex (PLR) is elicited upon illumination of the eyes, protecting the retina from excess light exposure. The neural pathway inducing the PLR starts from the retinal photoreceptors and intrinsically photosensitive retinal ganglion cells (ipRGCs), carrying signals to the olivary pretectal nucleus (OPN) and Edinger–Westphal nuclei in the midbrain, finally returning to the ciliary ganglion in the orbit and constricting the pupil. Because the PLR is easily examined, it has been broadly used in the clinic for diagnosing eye and brain diseases, such as glaucoma and brain injury (La Morgia et al., 2018; Pinheiro and Da Costa, 2021).

ipRGCs were identified relatively recently. Lucas et al. (2001, 2003) reported that mice with rod/cone degeneration (rd/rd) retain a robust PLR at high irradiances. Further elimination of the melanopsin gene in these mice diminished PLR (Lucas et al., 2003), indicating that melanopsin-expressing cells directly sense light and induce the PLR. Melanopsin-expressing cells are categorized as ipRGCs, and have been shown to be crucial in non-image-forming vision, including PLR and circadian photoentrainment (Hattar et al., 2003; Guler et al., 2008; Aranda and Schmidt, 2021). rd/rd mice do not show PLR in scotopic-to-mesopic light (Lucas et al., 2001; Lucas et al., 2003), suggesting that rod/cone photoreceptor inputs to the ipRGCs induce PLR in lower light conditions, while melanopsin is responsible for PLR at higher irradiances.

In conjunction with melanopsin photosensitivity, ipRGCs receive rod/cone photoreceptor inputs. The roles of rods/cones and melanopsin responses in PLR have been investigated using mutant mouse models. In rod/cone dysfunctional mouse and human models, PLR was evoked at high irradiances but with significantly slower onset than in the control subjects (Gooley et al., 2012; Kostic et al., 2016). The PLR onset delay was also observed in mGluR6-KO mice, where rod/cone signaling was not properly transferred to ON bipolar cells and downstream ganglion cells (Beier et al., 2022). On the contrary, in mice lacking melanopsin, due to an elimination of opn4 gene (opn4^–/–^), PLR was induced across a wide range of irradiances; however, the pupil constriction occurred only transiently during the light exposure (Zhu et al., 2007). These reports indicate that rod/cone photoreceptors are crucial for PLR at all stimulus intensities to induce rapid PLR onset, whereas melanopsin response in ipRGCs induces PLR only at high irradiance to shape the sustained components of PLR.

Most of the studies on PLR have been conducted using transgenic mouse models, and we wondered whether those mouse retinas might have compensatory effects or changes by remodeling. We used N-nitroso-N-methylurea (MNU) to selectively ablate rod/cone photoreceptors in a short span of 5–7 days (Smith and Yielding, 1986; Smith et al., 1988), a method that minimizes the potential for plastic changes. This approach allowed us to examine the roles of rod/cone photoreceptors and ipRGCs in the PLR, revealing features of ipRGCs that were unknown thus far.

## Materials and methods

### Animal model set-up

All experimental procedures with animals were approved by the Institutional Animal Care and Use Committee at Wayne State University (IACUC 20-10-2909 & 23-11-6310). Experiments were performed in accordance with the ARVO Statement for the Use of Animals in Ophthalmic and Visual Research. Wildtype (WT) (C57BL/6J; #000664, Jackson Laboratory, ME, United States), ranging from 1 to 6 months of age in both sexes were used in this study.

N-nitroso-N-methylurea (MNU) mice were generated as follows: MNU solution (HY-34758, MedChem Express, NJ, Unite States) was injected intraperitoneally (i.p.) into WT mice with a single dose at 62.5–93.75 mg/kg. Pupillometry and MEA recordings were conducted after 5–7 days of injection. As a control, saline was i.p., injected to WT mice.

Rod/cone double knockout (Cnga3^–/–^, Gnat1^–/–^; dKO) mice (gifted by Dr. Samar Hattar) that lack the cone cyclic nucleotide-gated channels (Cnga3) and rod transducin α-subunit (Gnat1), crucial molecules for the visual cycle in photoreceptors. dKO mice were evaluated for PLR, MEA, and IHC staining. Heterozygous littermates were used as controls (Cnga3^+/–^, Gnat1^+/–^; dKO^+/–^).

### Pupillometry

Mice were dark adapted for over an hour prior to PLR measurements. Recordings were performed between 3 and 6 Zeitgeber time. For all recordings, mice were mechanically restrained by an experimenter’s hand for a few minutes during the pupillometry. No anesthesia was used. The pupillometry was conducted from one or two eyes in each mouse. For two eye measurements, after the completion of the first eye measurement, we dark adapted the mouse for 30 min before measuring the second eye. We conducted pupillometry only once per mouse. All procedures were conducted in dim red-light conditions.

The pupillometry was conducted using a stereo microscope equipped with a fixed-focus video camera (Lumenara 400; Teledyne Technologies, CA, United States) under illumination with an 850 nm infrared LED (M850L3; ThorLabs, NJ, United States). PLR was induced by 500 nm (green) light for 10 s at three stimulus intensities: low (mesopic, 5.34 × 10^4^ photons/μm^2^/s), medium (photopic, 2.95 × 10^5^ photons/μm^2^/s), and high (high photopic, 3.72 × 10^6^ photons/μm^2^/s). These intensities were measured from where mouse heads were placed under the LED using a photometer (International Light Technologies, Peabody, MA, United States). PLR recordings were video recorded at ∼25 frames per second (FPS) (NorPix 9; NorPix, Quebec, CA, United States).

### Retinal preparation

The experimental techniques were similar to those described previously (Ichinose et al., 2014; Ichinose and Hellmer, 2016). Briefly, mice aged 28–60 days were euthanized and the wholemount retinal preparations or 250-μm-thick slice preparations were made in dark conditions using oxygenated HEPES-buffered solution containing the following (in mM): 115 NaCl, 2.5 KCl, 2.5 CaCl_2_, 1.0 MgCl_2_, 10 HEPES, and 28 glucose, adjusted to pH 7.37 by NaOH.

### Immunohistochemistry

We used our standard procedure for immunohistochemistry (Farshi et al., 2016). The retinal wholemount preparations were fixed using 4% paraformaldehyde and blocked with 10% normal donkey serum (NDS) and 0.5% Triton-X in PBS (PBS-T). Melanopsin antibody (1:5000, AB-N39, Advanced Targeting Systems, CA, United States) in PBS-T was incubated for 3 days at 4°C, followed by Alexa568 donkey-anti-rabbit (Invitrogen; A10042) for 2 h. Gnat1 antibody (1:1000, PA5-28336, Thermo Fisher) was incubated overnight at RT. DAPI (1:10000, Sigma, Co.) was applied for 20 min along with the secondary antibody incubation. Stained tissue was viewed with a confocal microscope (TCS SP8; Leica, DE). Using 40× objective lens, either the photoreceptor or ganglion cell layer was fully scanned at a digital step of 0.3 μm. Confocal images were captured in the middle of peripheral retina for all four quadrants of the retina.

### Multi-electrode array (MEA)

Wildtype (WT), MNU, and dKO mice were dark adapted overnight and retinal wholemount preparations were prepared under infrared light using night vision devices. The retinal preparation was then divided into 4 quadrants. Each retinal quadrant was placed onto an Accura HD-MEA chip (4,096 recording electrodes arranged in a 3.8 mm × 3.8 mm area multielectrode array) for the BioCAM DupleX system (3Brain, Switzerland) with a piece of clear filter paper, immobilized by a square platinum anchor. The preparation was continuously perfused with oxygenated AMES’ medium at a rate of 3–7 ml/min and maintained at 33–34°C with a temperature controller (Warner TC-324C, ALA Scientific, NY, United States). In some recordings, a cocktail of glutamate receptor blockers was applied to block photoreceptor synaptic inputs to the retinal neural networks that include: an mGluR6 agonist, L-2-amino-4-phosphonobutyric acid (L-AP4) (10 μM), an ionotropic glutamate antagonist, 6-cyano-7-nitroquinoxaline-2,3-dione (CNQX) (15 μM), a kainite antagonist, (S)-1-(2-Amino-2-carboxyethyl)-3-(2-carboxy-5-phenylthiophene-3-yl-methyl)-5-methylpyrimidine-2,4-dione (ACET) (1 μM), and an N-methyl D-aspartate (NMDA) antagonist, D-(-)-2-Amino-5-phosphonopentanoic acid (D-AP5) (50 μM). All glutamate blockers were obtained from Tocris, R&D Systems Inc. (Minneapolis, MN). Light-evoked spike responses in ganglion cells were induced by green light stimuli of the same three intensities used for PLR measurements.

### Data analysis

Pupillary light reflex (PLR) videos were analyzed using AIVIA software (Leica) to measure pupil diameter in each frame during recordings. PLR constriction curves were fitted with a single component exponential function using SigmaPlot15 (Grafitti, CA, United States) (*R*^2^> 0.90 for fitted curves), and the latency, curvature (tau), and peak constriction were compared by a one-way ANOVA using Tukey’s method *post-hoc* analysis for comparisons between WT, MNU-injected, and dKO mice. For all statistical measures, n equaled number of eyes, and a *p*-value < 0.05 was considered significant.

For the immunohistochemistry images, melanopsin and DAPI cell counting was performed using ImageJ (NIH, MD, United States). Gnat1 and DAPI stained photoreceptors were analyzed using the AIVIA software. For each condition, cells were analyzed in an image size of 140 × 140 μm for each quadrant of the retina. One-way ANOVA using Tukey’s method for *post-hoc* comparisons was used to compare DAPI and melanopsin cell counts in the saline, MNU injected, and dKO mice. For all analyses, *p* < 0.05 was considered to be significant.

The MEA analysis was conducted using the Brainwave 5 software (3Brain, Switzerland), and customized Python and MATLAB codes (a generous gift by Dr. Benjamin Sivyer). An automated spike sorter^1^ was used to detect and cluster spikes based on location and two components of waveform as described in Hilgen et al. (2017). Multiple trials for each tissue were sorted separately, and similar cluster numbers were found for each case. Further analysis on the spike trains and firing rates (FR) was performed using MATLAB (MathWorks, Natick, MA, United States). For each trial, all detected units underwent the same unbiased filtering. ipRGC units were defined by three parameters based on previous studies (Qiu et al., 2005; Prigge et al., 2016; Mure et al., 2019; Berry et al., 2023): the mean firing rate (mFR) reached baseline mFR + 2 × SD (standard deviation) after the start of stimulation (response latency); the mFR for a 20 s period after this threshold stayed baseline mFR + SD; and the mFR for a period after stimulus offset was greater than baseline mFR + SD. Cell units that never reached greater than baseline mFR + 2 × SD or that had zero baseline mFR were discarded. We defined the fast-onset ipRGC units as ones that had a response latency between 0 and 2 s after the start of the stimulus.

Curve fitting was done using SigmaPlot15.0 (Grafitti) to calculate the rising phase rate. An exponential rise function was applied: y = ae^bt^

where (a) is the baseline mFR, while (b) is the growth rate (if *b* > 0 means exponential growth, *b* < 0 means exponential decay), (e) is the base of natural logarithm and (t) denotes time. We calculated the firing rate increase (b value) in response to light for each retinal tissue. This process was carried out for each mouse type and subsequent comparisons were made. Furthermore, latency time was measured between the light stimulus onset and the time at the 63% of the rate peak. ANOVA was utilized to compare these values in Prism 8 (GraphPad Software Inc., CA). A *p*-value < 0.05 was considered significant for this analysis.

## Results

### PLR from C57 wildtype, MNU, and rod-cone dysfunctional mice

**FIGURE 1:**
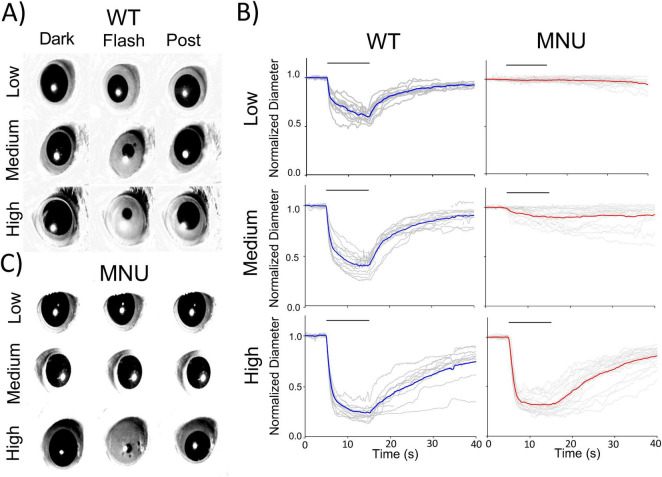
Pupillary light reflex (PLR) in wildtype (WT) and N-nitroso-N-methylurea (MNU)-injected mice: **(A)** Single frame images from PLR recordings for WT mice. Pupil images before the light stimulus (Dark), during the light stimulus (Flash), and the recovery phase (Post) are presented for each light intensity. **(B)** Normalized pupil diameters were plotted as a function of time. Individual eyes (gray traces) and average pupil constriction for WT (blue) and MNU (red) were overlaid. **(C)** A set of representative pupil images for MNU-injected mice. PLR was barely detected in response to low and medium light stimuli. In contrast, high light evoked PLR, and the kinetics and levels were similar to WT PLR.

We conducted PLR in WT mice to examine retinal cellular contributions. Dark adaptation fully dilated the pupil before light exposure. In response to a low light flash for 10 s, pupils slightly constricted and recovered (Figures 1A, B, Low). As irradiance increased, pupil constriction was faster and more robust (Figures 1A, B, Medium and High).

The PLR is mediated by ipRGCs (Guler et al., 2008), which sense light directly through melanopsin (Lucas et al., 2003), as well as receive synaptic inputs from photoreceptors. To examine the roles of melanopsin and photoreceptors in PLR, we injected MNU (i.p.) into WT mice, which has been shown to eliminate photoreceptors within several days, and thus, PLR is evoked only by melanopsin response (Smith and Yielding, 1986; Smith et al., 1988; Wang et al., 2021). Five to seven days after MNU injection, we measured the PLR (Figures 1B, C). The low-light stimulus did not evoke PLR in MNU mice, suggesting that rods and cones are essential for PLR at this light condition. The Medium light stimulus evoked the PLR in MNU mice, but with a smaller constriction and slower time course than WT mice (Figures 1B, C, medium). These results are consistent with previous observations, showing ipRGC’s low sensitivity and sluggish melanopsin responses (Gooley et al., 2012; Kostic et al., 2016; Beier et al., 2022). We then increased the stimulus intensity to high irradiance. PLR was evoked fully and rapidly, identical to the PLR in WT mice (Figure 1B, High). This result was unexpected because previous work concluded that photoreceptors shape the PLR onset at all the irradiances (Gooley et al., 2012; Kostic et al., 2016; Beier et al., 2022).

**FIGURE 2:**
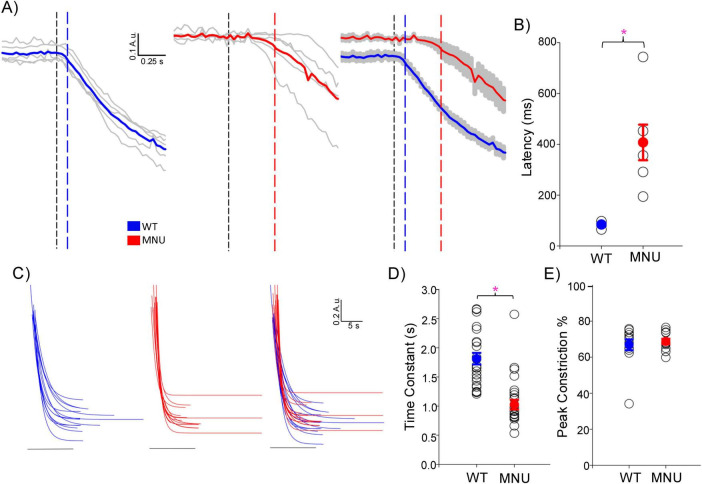
The initial phase of high light-evoked pupillary light reflex (PLRs) was equivalent between the wildtype (WT) and N-nitroso-N-methylurea (MNU) eyes: **(A)** the initial phase is displayed in a short time scale for WT (blue) and MNU eyes (red). The black dotted vertical line indicates the light stimulus onset, and either blue or red line indicates the PLR starting point. The latency from the light onset to the PLR start was shorter in WT than in MNU eyes. **(B)** A summary graph shows the latency in WT and MNU eyes, which showed a significant delay in MNU eyes (*p* < 0.05, Student’s unpaired *t*-test). The means and SEM for WT and MNU are presented in blue and red, respectively. **(C)** The initial phases of high light-evoked PLRs were fit with single component exponential decay curves for WT eyes (blue) and MNU (red) mice (*R*^2^> 0.90). **(D)** A graph showing each curve fit’s time constant (tau). MNU exhibited low tau, indicating a faster PLR than WT PLR (*p* < 0.05, *n* = 12 mice). **(E)** A graph showing peak constriction showed no differences between WT and MNU mice (*p* = 0.07, *n* = 12 mice). *p < 0.05.

We analyzed the high light-evoked PLR onset in WT and MNU eyes (Figure 2). We found that the PLR in WT eyes showed significantly shorter latency than in MNU eyes (Figures 2A, B, *n* = 5 WT and *n* = 5 MNU, *p* < 0.05, unpaired Student’s *t*-test). However, the speed of PLR constriction was faster in MNU than in WT eyes (Figures 2C, D, *p* < 0.05, paired *t*-test, *n* = 21 eyes), reaching peak constriction at similar timing (Figure 1, High). The level of constriction was the same between WT and MNU eyes (Figure 2E, *p* = 0.94). These analyses confirmed that the rising phase of high light-evoked PLR in MNU mice was equivalent to the WT eyes. Because the MNU eyes contain only ipRGCs, our results indicate that ipRGCs fully contribute to PLR at this light stimulus.

Previous reports used mutant mouse models to examine the role of ipRGCs in PLR, demonstrating a slower and smaller PLR in these mice at any stimulus light intensities (Gooley et al., 2012; Kostic et al., 2016; Beier et al., 2022). Our MNU results were contradictory to these reports (Figure 1). To compare PLR between our MNU model and mutant mouse models, we measured the PLR in a mouse model with dysfunctional rod-cone photoreceptors (Cnga3^–/–^ & Gnat1^–/–^: dKO). After dark adaptation, their pupil sizes fully dilated, which did not differ from WT mice (one-way ANOVA, *p* > 0.5).

**FIGURE 3:**
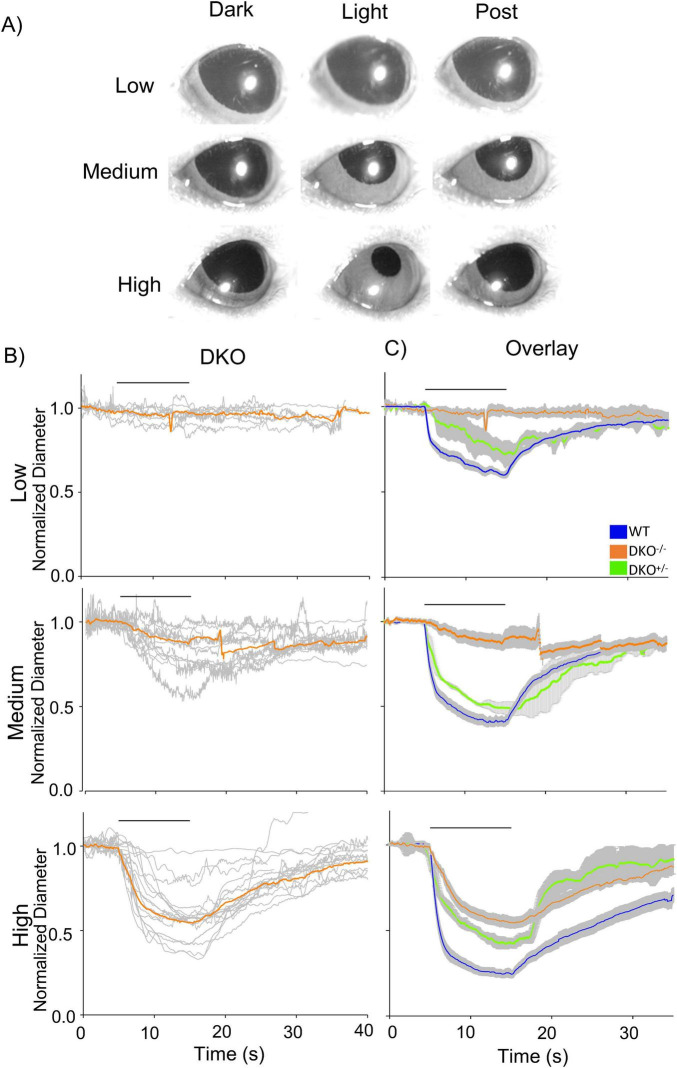
Pupillary light reflex (PLR) in dKO mice. (A) A set of pupil images showing PLR from dKO mice. No PLR was induced by low light, and small constrictions were evoked by medium- to high-light stimuli. (B) Normalized pupil diameters over time were plotted from dKO mice (n = 10). The individual dKO eyes were in gray and the average responses of all eyes were in orange. (C) The mean PLRs for dKO eyes (orange), littermate control for dKO (dKO^+/–^: green), and wildtype (WT) (blue) are overlaid with the S.E.M. (gray).

The low-light stimulus minimally evoked PLR in dKO mice, and medium-light stimulus evoked small and slow PLR (Figure 3B, Low and Medium), similar to our MNU mice. The high-light stimulus evoked a more robust response (Figure 3B, High); however, the level of pupil constriction was smaller than the WT PLR, and the PLR onset was slower (*p* < 0.0001, Figure 3C, High). We compared the results with their littermate controls (Cnga3^+/–^ & Gnat1^+/–^; dKO^+/–^, *n* = 3 mice). Their PLRs were similar to the WT for low and medium light (Figure 3C, green). However, high light did not evoke the PLR fully to the level of the WT, suggesting that the smaller and slower PLR observed in the dKO mice was attributed to this mouse strain. Even though both MNU and dKO mice respond to light using ipRGCs alone, MNU mice showed a much faster constriction in response to high light stimuli comparable to WT mice.

### Photoreceptors were dysfunctional in MNU retinal tissue

**FIGURE 4:**
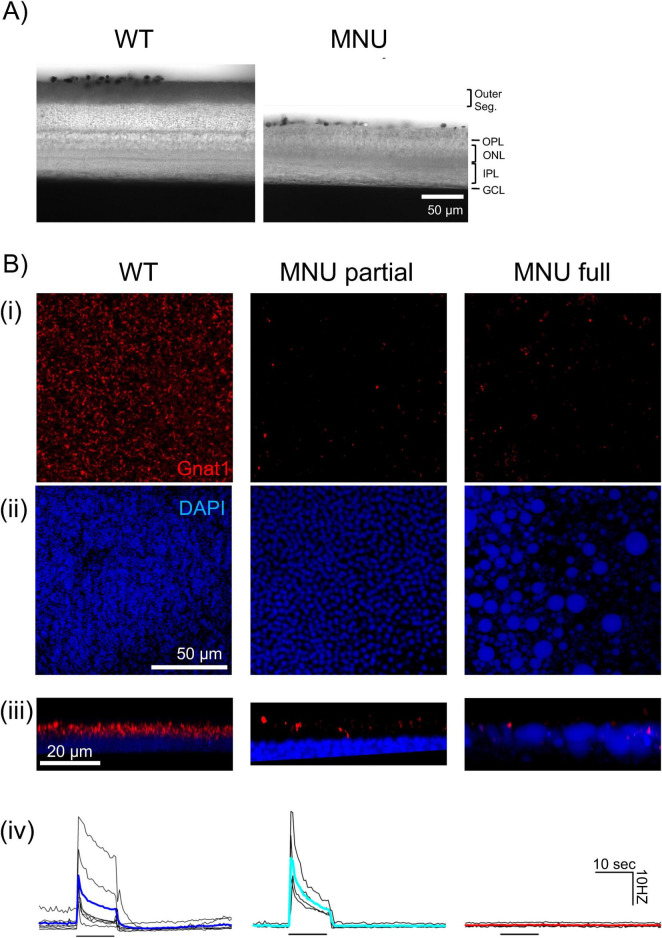
N-nitroso-N-methylurea (MNU) selectively ablated rod/cone photoreceptors: **(A)** DIC images showing retinal vertical sections from wildtype (WT) (left) and MNU eyes (right). MNU-injected retinas showed a loss of the photoreceptor layer, including the outer segment and outer nuclear layer. The inner retinal layers remained intact. **(B)** Immunohistochemistry images of the photoreceptor layer in wholemount tissues panel (i–iii). (Left column) The WT mice without MNU revealed a massive Gnat1-labeled rod outer segment (red) and DAPI-labeled photoreceptor somas (blue). The lower panel (iii) shows a digitally rendered image of the side view. The lowest panel (iv) individual traces (black) and the mean (blue) showing the MEA recording of ganglion cell spikes in response to a low-light stimulus. (Middle column) The MNU partial tissue shows the decreased Gnat1 puncta with almost intact somas. The MEA recording [individual traces (black) and the mean (cyan)] showed low light-evoked light responses panel (iv). (Right column) In general, MNU injection almost abolished the Gnat1-labeled outer segment panel (i), which resided in the DAPI-labeled soma layer panel (iii). The somas were swollen and exhibited cobblestone-like irregular structures panel (ii). The side view revealed the swollen somas with intermingled Gnat1 puncta. For these eyes, MEA recording individual traces (black) and the mean (red) low light did not evoke ganglion cell spikes panel (iv).

It was shown previously that photoreceptors remained after MNU injection (Jain et al., 2016; Tao et al., 2015), which might have induced the fast ipRGC response in our MNU mice. We conducted tissue analysis to examine whether MNU injection removed photoreceptors. We first compared vertical retinal sections (Figure 4A). The MNU injection eliminated the outer retinal layers, from the outer plexiform layer (OPL) to photoreceptor outer segments (Figure 4A, *n* = 6 mice for saline, 12 mice for MNU). Retinal sections were taken from both the central and peripheral retina; photoreceptor loss was uniform across the retina after MNU injection.

However, degenerating photoreceptors could respond to light in rd10 mice in which the majority of rods and cones were eliminated (Ellis et al., 2023). We examined our MNU tissues using the anti-Gnat1 antibody and DAPI staining [Figure 4B panel (i–iii)]. In WT tissues, Gnat1 stained the outer segments of rods and DAPI stained the photoreceptor somas ([Figure 4B panel (i), “WT”]. The side view of the photoreceptor layer showed that somas and outer segments were polarized [Figure 4B panel (iii), “WT”]. We conducted MEA using the retinal tissue from the same eyes and found that many ganglion cells responded to a low light stimulus [Figure 4B panel (iv), “WT”].

When MNU was injected, Gnat1 staining was significantly reduced (WT: 185,460 ± 31,900 rods/mm^2^, *n* = 3; partial MNU:16,980 ± 46,300 rods/mm^2^, *n* = 7, *p* < 0.05, unpaired Student’s *t*-test). However, in some cases, a low-light stimulus still evoked MEA light responses similarly, to WT tissues [Figure 4B panel (iv), “MNU partial”]. In these cases, although Gnat1 staining was significantly reduced, their somas and the soma-outer segment polarization were preserved [Figure 4B panel (iii), “MNU partial”].

However, in most cases, MNU injection eliminated low light-evoked responses in MEA recordings [Figure 4B panel (iv), “MNU full”]. Gnat1 staining was significantly reduced compared to WT and MNU partial (1,190 ± 700 rods/mm^2^, *n* = 4, *p* < 0.05 vs. control, unpaired Student *t*-test). Furthermore, photoreceptor somas were swollen, and the soma outer segment polarization disappeared [Figure 4B panel (ii), “MNU full”].

Based on these observations, we considered MNU was fully effective when the low light-evoked response disappeared. When light response in MEA recordings or PLR was evoked by low light even after MNU injection, we considered that the MNU was partially effective and excluded those mice from our MNU group.

**FIGURE 5:**
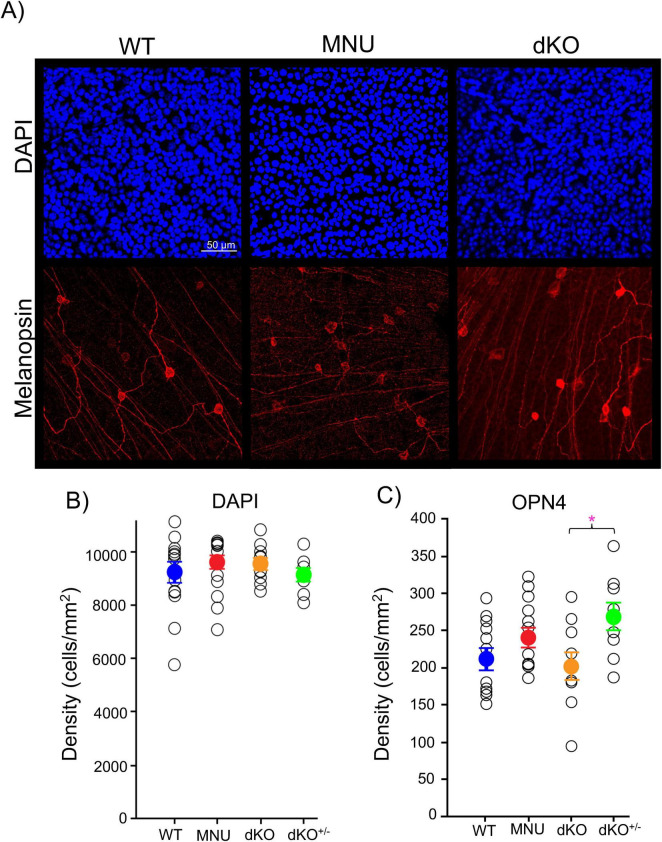
Ganglion cells were intact in N-nitroso-N-methylurea (MNU) and dKO mice: **(A)** Representative ganglion cell layer images after immunostaining with DAPI (blue) and OPN4 intrinsically photosensitive retinal ganglion cells (ipRGCs, red) in the wildtype (WT), MNU, and dKO retinal tissues. **(B)** The number of ganglion cell layer cells (GCL) somas by DAPI staining remained similar between the WT (blue), MNU (red), dKO (orange), and dKO^+/–^ (green) retinas (*p* = 0.385, *n* = 15 WT, *n* = 17 MNU, *n* = 9 dKO^+/–^, and *n* = 10 dKO mice, one-way ANOVA with Tukey’s Method *post-hoc* comparisons). **(C)** The number of ipRGCs also remained constant in WT, MNU, and dKO^+/–^ mice; however, dKO showed a decreased in comparison to their heterozygous littermates (*p* = 0.022, *n* = 12 WT, *n* = 13 MNU, *n* = 9 dKO^+/–^, and *n* = 10 dKO mice. One-way ANOVA with Tukey’s *post-hoc* comparisons). *p < 0.05.

We also examined whether MNU affected the ganglion cell layer cells (GCL) (Figure 5A). The GCL structure was examined by immunohistochemistry with DAPI and the opn4 antibody, which stained the GCL nuclei and ipRGCs, respectively. We found that the number of cells in the GCL was similar between WT and MNU tissues (Figures 5A, B, *p* = 0.29, *n* = 10 WT and *n* = 11 MNU). The number of ipRGCs varied among retinas. Although the average ipRGC number was the same in the WT, MNU, and dKO retinal tissues, dKO^+/–^ mice showed a larger number of ipRGCs than dKO mice (Figures 5A, C, *p* = 0.022, one-way ANOVA, *n* = 12 WT, *n* = 9 dKO^+/–^, *n* = 13 MNU, and *n* = 10 dKO). This implies minor remodeling in these mice. Nevertheless, the number of GCL cells remained consistent across experimental conditions (Figure 5B, *p* = 0.56, one-way ANOVA). These results indicate that the GCL remodeling minimally occurred in these mice, and the fast onset of high light-induced PLRs in MNU mice was not attributable to compensatory increased ipRGCs.

### ipRGC light-evoked response recording by MEA

**FIGURE 6:**
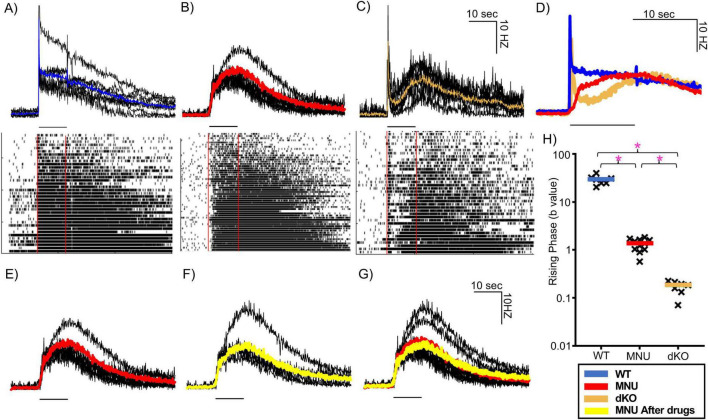
MEA recording from intrinsically photosensitive retinal ganglion cells (ipRGCs) in three mouse types in response to high-light stimuli. **(A–C)** Individual trace in black indicates ipRGC units in wildtype (WT) **(A)**, N-nitroso-N-methylurea (MNU) **(B)**, and dKO **(C)**, and average traces are shown in color (WT: blue, *n* = 7 tissues, MNU (red), *n* = 11 tissues, dKO (orange), *n* = 7 tissues). Black lines under each trace indicates the 10 s light stimulus timing. Lower panels show raster plots containing individual spikes of a representative population of ipRGCs from each respective tissue. **(D)** The ipRGC average spike rate traces of the three different mouse types are overlaid. Three colors indicate three mouse strains as shown in panels **(A–C)**. **(E–G)** The average spike rate of MNU injected mice at high-light intensity (red) **(E)** and in the presence glutamate receptor blockers (L-AP4, D-AP5, ACET and CNQX) (yellow) **(F)**. **(G)** An overlay of these traces, showing no changes in ipRGC spikes. **(H)** A column chart showing the curvature (b) of the initial phase of ipRGC spiking rate from three different mouse types. The black crosses indicate individual traces, while the colored bar indicates the mean of all the traces of each type. *p < 0.05.

To explore the physiological mechanisms underlying the differential PLR responses in WT, MNU, and dKO mice, we used MEA to record light-evoked responses in ipRGCs. We evoked ipRGC spike rate responses using a high light stimulus—the same “high light” intensity used for PLR recordings. Hundreds to thousands of units were detected per tissue and we separated ipRGC units based on the criteria described in the Methods section. In WT tissues, ipRGC units were defined by having spikes during the light onset and long-lasting responses (Figure 6A, lower panel). These units showed light-evoked responses immediately after light stimulus, indicating photoreceptor inputs in WT mice (Figure 6A, *n* = 4 WT mice).

In MNU-injected retinal tissues (n = 11 tissues from eight mice), a low light stimulus did not evoke light responses [Figure 4B panel (iv)]. In contrast, the high light stimulus evoked light responses with long-lasting spikes, suggestive of ipRGCs. We found that a subset of ipRGC units responded immediately after the light onset. Shown in every MNU tissue (mean: 15.6%, range 6.7–23.3% of ipRGC units, Figure 6B). The latency between the light onset and at 63% of the peak response was 1.57 ± 0.07 s (*n* = 4 MNU tissues), which was comparable to the time of PLR in Figure 2 (latency + time constant: 1.5–2 s).

We recorded the MEA with dKO mouse tissues to examine whether the population of fast-responding ipRGCs found in the MNU tissues at high light were present (Figure 6C, *n* = 4 dKO mice). In response to the light stimulus, an initial transient response was evoked that quickly subsided followed by a slow-onset, long-lasting response (Figure 6C). The initial response was most likely induced by reminiscent Gnat2 in rod photoreceptors and not by ipRGCs (Allen et al., 2010). ipRGCs induced the slow-onset, long-lasting response. We did not find any fast-onset ipRGC units that matched the kinetics of those in the MNU retinas (Figures 6B, C). Figure 6D overlays average ipRGC responses from WT, MNU, and dKO tissues, demonstrating that the dKO spiking response had a significantly slower ipRGC component onset than both WT and MNU tissues. To analyze the onset of ipRGC response in these tissues, we used the curve fitting property to obtain the (b) value, which represents the curvature of the rising phase (refer to Methods section for details). A graph of these (b) values (Figure 6H) revealed that the WT tissue rising phase was faster than MNU and dKO (*p* < 0.0001, one-way ANOVA), and the MNU tissue exhibited a faster rising phase than the dKO tissue (*p* < 0.0001, one-way ANOVA). These temporal results are consistent with our PLR results (Figures 1, 3), suggesting that some ipRGCs respond to light fast, to shape the initial component of PLRs in MNU tissue which was missing in the mutant mice.

To further rule out any involvement of photoreceptors input in the fast response of MNU mice, we applied a cocktail of glutamate receptor blockers to suppress synaptic transmission from photoreceptors to ipRGCs (*n* = 5 tissues from four mice). We recorded spike rates before and after perfusion of glutamate receptor blockers (Figures 6E, F). When overlaid, no difference was detected in time to reach 10% of the peak (before: 0.55 ± 0.03 s, after: 0.525 ± 0.09 s, *n* = 5 *p* = 0.9, unpaired Student’s *t*-test) and peak spiking rate (before: 16 ± 1.6 Hz, after: 13.8 ± 2.3 Hz, *n* = 5, *p* = 0.4, unpaired Student’s *t*-test) (Figure 6G), indicating that photoreceptor-evoked inputs did not contribute to the response. This result along with the evidence that no response was elicited by the low light stimulus (Figure 4B) confirms that photoreceptor inputs were eliminated in MNU mice.

## Discussion

We examined the roles of rod and cone photoreceptors and melanopsin in PLR by MNU injection to eliminate photoreceptors in 5–7 days. Although MNU mice showed reduced PLRs in response to low and medium light stimuli, a high light stimulus unexpectedly evoked fast and robust PLR, equivalent to WT mouse eyes. We did not observe robust PLR in the dKO mutant mice. We then used retinal *ex vivo* preparations to examine the ipRGC spikes using MEA, which exhibited that approximately 15% of ipRGCs in the MNU tissue had fast onset. Our live mice and *ex vivo* MEA results indicate that the intrinsic light responses in ipRGCs are capable of inducing PLR using a highlight stimulus, which contradicts the previous reports showing the crucial role of photoreceptors in high light-induced PLR.

### MNU injection and photoreceptors/ipRGCs

N-nitroso-N-methylurea (MNU) is a DNA alkylating agent distributed widely in the environment as a potent carcinogen (Smith et al., 1988) and has been used to produce a retinal degeneration mouse model, which eliminates the photoreceptors without affecting inner retinal neurons (Kiuchi et al., 2002; Wang et al., 2021). DNA alkylating defect and subsequent glycosylase (AAG) and poly (ADP-ribose) polymerase (PARP1) activation for apoptosis pathway occur only in photoreceptors, sparing inner retinal neurons (Calvo et al., 2013; Meira et al., 2009; Uehara et al., 2006).

Consistent with these previous reports, we observed that the retinal photoreceptor layers were eliminated only 5–7 days after MNU injection in all tested mice (Figure 4A). Although we observed that the photoreceptor outer segment was eliminated from the central to peripheral regions, reminiscent photoreceptors may still respond to a high light stimulus. Jain et al. (2016) conducted immunostaining with s-opsin, m-opsin, and rhodopsin and observed reminiscent photoreceptors after 7 days of MNU injection. Tao et al. (2015) observed that MNU eliminated rods in 7 days but cone elimination took 10 days. Furthermore, in rd10 mice, degenerating photoreceptors continued to respond to light even with a shortened outer segment (Ellis et al., 2023). Therefore, we examined reminiscent photoreceptors in the MNU tissue.

The MNU injection drastically reduced the photoreceptor layer and Gnat1-stained puncta after 5–7 days of MNU injection (Figure 4). In some MNU tissues, photoreceptor somas were still observed, and photoreceptor-evoked responses were still elicited by low light stimuli in same eyes (Figure 4B, “MNU partial”). However, in most MNU tissues, low light-evoked responses were not observed (Figure 4B “MNU full”). Furthermore, the high light stimulus evoked ipRGC responses but never evoked cone photoreceptor signaling at the offset of light stimulus (*n* = 11 tissues). In these same tissues, we observed swollen somas near the OPL with small numbers of Gnat1 puncta hovering. These results indicate that both rod and cone photoreceptors were no longer functional in these retinas after 5–7 days of MNU injection. We used the latter as MNU mice for both PLR and MEA recordings, ensuring that the fast and robust PLR and ipRGC responses in MNU mice were not attributable to photoreceptor responses.

Even though photoreceptors were eliminated in the MNU mice and were dysfunctional in the dKO mice, ganglion cells were intact (Figure 5). Jain et al. (2016) observed increased ipRGCs in the MNU tissue. The range in the number of ipRGCs was relatively large (Figure 5C), and the number of ipRGCs might be increased after MNU injection in some cases. However, it is unlikely that the increased number of ipRGCs in MNU eyes make new connections with postsynaptic targets within several days to boost the PLR. Taken together, we confirmed that MNU injection eliminated photoreceptors over 5–7 days without affecting ipRGCs.

### Contribution of rod/cone photoreceptors and ipRGCs to PLR

Intrinsically photosensitive retinal ganglion cells (ipRGCs) are crucial neurons for non-image-forming vision, that also receive synaptic inputs from rod/cone photoreceptors. The roles of these photosensitive cells in the PLR have been investigated primarily in photoreceptor degeneration mouse models. Since rod photoreceptors are more sensitive to light than cones and ipRGCs (Field and Rieke, 2002; Pang et al., 2004; Qiu et al., 2005 Berson et al., 2002), rods are crucial for low light-evoked PLR. A photoreceptor degeneration model—rd/rd cl mice—showed PLR only at high-irradiances, but not low- to mid-irradiances (Lucas et al., 2001; Lucas et al., 2003). In these mice, PLR was induced by light stimulus at an intensity equivalent to the operational range of the ipRGCs (Lucas et al., 2001). PLRs in the MNU and dKO mice in our experiments were consistent with those of the rd/rd mice that did not show PLR up to an irradiance of 10^4^ photons/μm^2^/s (or 10^12^ photons/cm^2^/s), which was our low light stimulus intensity (Figures 1–3).

The ipRGCs exhibit low sensitivity to light stimuli with sluggish and long-lasting responses (Berson et al., 2002; Dacey et al., 2005). Because of this slow nature, differential temporal roles of these photosensitive cells have been explored. Lucas et al. (2001) compared the high light-evoked PLR in wildtype and rd/rd cl mice and found that the onset of PLR was significantly delayed compared to the WT. Kostic et al. (2016) used rod/cone dysfunctional mice (Cnga3^–/–^; Rho^–/–^ or Gnat1^–/–^) to reveal a small and slow PLR. Similarly, Beier et al. (2022) used mGluR6-KO, Elfn1-KO, and Cone-Cx36-KO mice in which photoreceptor transmission to third-order neurons are disconnected to conclude that ipRGCs shape the sustained phase of the PLR. A study with blind humans, whose photoreceptors were dysfunctional but whose circadian rhythms were normal, exhibited similar results (Gooley et al., 2012). On the other hand, the opn4^–/–^ mice exhibit short-lasting PLR (Zhu et al., 2007). All these results demonstrate that photoreceptors are crucial for initiating PLR even at the high light stimulus, and melanopsin serves for sustained response during light exposure. Our dKO mice (Cnga3^–/–^; Gnat1^–/–^) showed consistent results (Figure 3).

Nevertheless, our MNU mice did not show the same results. Although PLR onset was delayed in MNU mice (Figures 2A, B), probably due to the photoreceptor elimination, the constriction speed in MNU eyes was faster than those in WT mice (Figures 2C, D), reaching peak constriction with similar timing between the two conditions (Figure 1). Based on the measurements in Figure 2, the overall PLR time to 63% peak was approximately 1.5–2.0 s for both conditions (latency + time constant). Our MEA recordings support the PLR results. In MNU mice, we found that approximately 15% of ipRGCs generated spikes with fast onset: the time to 63% of peak was 1.5 s on average for MNU tissues. This fast ipRGC light response is not likely attributable to the remodeling because the number of DAPI-stained ganglion cell layer cells and the OPN4-expressing ganglion cells was the same among WT, MNU, and dKO mice (Figure 5).

The primary difference between the previous work and ours is the mouse models. Most of the previous work used mutant mouse models, including photoreceptor degenerating models (rd/rd), photoreceptor dysfunctional models (Cnga3-KO, Gnat1-KO), and retinal network mutants (mGluR6-KO). For instance, although the dKO tissue had an initial increase in spiking owing to reminiscent rod activity, it was transient and did not speed up the overall response of the ipRGCs. In contrast, we injected MNU to eliminate photoreceptors over several days, limiting the risk of remodeling and compensatory effects. This way, we revealed that ipRGCs responded fast enough to shape high light-evoked PLR without photoreceptors. Our novel observation of the contribution of ipRGCs to PLR might be crucial for diagnosing eye disease in clinical situations. Furthermore, we believe our observation will contribute to a more complete understanding of retinal circuitry from phototransduction to behavioral outcomes.

### Limitations of the study

Jain et al. (2016) used MNU to examine the cellular contributions to the PLR. Although we conducted similar procedures, our results and conclusions are not in agreement with theirs. In their MNU-injected mice, compared to the WT mice, pupil constriction in response to high light stimulus occurred only halfway, even though they observed an increased number of ipRGCs 7 days after MNU injection. Although the MNU concentration, mouse strains, and stimulus light intensities in their experiments were similar to ours, other conditions were distinct, such as anesthesia (ours: no anesthesia, Jain et al: light anesthesia) and dark adaptation before PLR stimuli [ours: > 30 min, Jain et al. (2016): 60–100 s], which might have caused the different outcomes.

We conducted PLR measurements *in vivo* and ipRGC recordings *ex vivo* to investigate whether ipRGC light response explains the PLR in MNU mice. We aligned the conditions such as light stimulus intensity and temperature in these measurements. However, these conditions cannot be the same between *in vivo* and *ex vivo* experiments. Light intensity at the retina in the *in vivo* mouse eye might be dimmer than in the MEA chamber during *ex vivo* studies because of light diffraction from the cornea and lens. The mouse body temperature (38°C) is higher than the MEA recording chamber (33–34°C) and could explain the low percentage of fast responding ipRGCs during these studies. Even though some conditions differed, we found that both recordings exhibited similar results in MNU tissue, demonstrating that ipRGCs’ direct light response explains the robust PRL in MNU mice.

## Data Availability

The original contributions presented in this study are included in this article/supplementary material, further inquiries can be directed to the corresponding author.

## Footnotes

1
https://github.com/mhhennig/HS2

## References

[B1] Allen A. E. Cameron M. A. Brown T. M. Vugler A. A. Lucas R. J. (2010). Visual responses in mice lacking critical components of all known retinal phototransduction cascades. *PLoS One* 5:e15063. 10.1371/journal.pone.0015063 21124780 PMC2993945

[B2] Aranda M. L. Schmidt T. M. (2021). Diversity of intrinsically photosensitive retinal ganglion cells: circuits and functions. *Cell Mol. Life Sci.* 78 889–907. 32965515 10.1007/s00018-020-03641-5 PMC8650628

[B3] Beier C. Bocchero U. Levy L. Zhang Z. Jin N. Massey S. C. (2022). Divergent outer retinal circuits drive image and non-image visual behaviors. *Cell Rep.* 39:111003. 10.1016/j.celrep.2022.111003 PMC9400924 35767957

[B4] Berry M. H. Leffler J. Allen C. N. Sivyer B. (2023). Functional subtypes of rodent melanopsin ganglion cells switch roles between night and day illumination. *bioRxiv [Preprint].* 10.1101/2023.08.26.554902 38168436 PMC10760181

[B5] Berson D. M. Dunn F. A. Takao M. (2002). Phototransduction by retinal ganglion cells that set the circadian clock. *Science* 295 1070–1073. 11834835 10.1126/science.1067262

[B6] Calvo J. A. Moroski-Erkul C. A. Lake A. Eichinger L. W. Shah D. Jhun I. (2013). Aag DNA glycosylase promotes alkylation-induced tissue damage mediated by Parp1. *PLoS Genet.* 9:e1003413. 10.1371/journal.pgen.1003413 23593019 PMC3617098

[B7] Dacey D. M. Liao H. W. Peterson B. B. Robinson F. R. Smith V. C. Pokorny J. (2005). Melanopsin-expressing ganglion cells in primate retina signal colour and irradiance and project to the LGN. *Nature* 433 749–754. 15716953 10.1038/nature03387

[B8] Ellis E. M. Paniagua A. E. Scalabrino M. L. Thapa M. Rathinavelu J. Jiao Y. (2023). Cones and cone pathways remain functional in advanced retinal degeneration. *Curr. Biol.* 33 1513–1522.e4. 36977418 10.1016/j.cub.2023.03.007 PMC10133175

[B9] Farshi P. Fyk-Kolodziej B. Krolewski D. M. Walker P. D. Ichinose T. (2016). Dopamine D1 receptor expression is bipolar cell type-specific in the mouse retina. *J. Comp. Neurol.* 524 2059–2079. 26587737 10.1002/cne.23932 PMC4860096

[B10] Field G. D. Rieke F. (2002). Nonlinear signal transfer from mouse rods to bipolar cells and implications for visual sensitivity. *Neuron* 34 773–785. 12062023 10.1016/s0896-6273(02)00700-6

[B11] Gooley J. J. Ho Mien I. St Hilaire M. A. Yeo S. C. Chua E. C. Van Reen E. (2012). Melanopsin and rod-cone photoreceptors play different roles in mediating pupillary light responses during exposure to continuous light in humans. *J. Neurosci.* 32 14242–14253. 23055493 10.1523/JNEUROSCI.1321-12.2012 PMC3515688

[B12] Guler A. D. Ecker J. L. Lall G. S. Haq S. Altimus C. M. Liao H. W. (2008). Melanopsin cells are the principal conduits for rod-cone input to non-image-forming vision. *Nature* 453 102–105. 18432195 10.1038/nature06829 PMC2871301

[B13] Hattar S. Lucas R. J. Mrosovsky N. Thompson S. Douglas R. H. Hankins M. W. (2003). Melanopsin and rod-cone photoreceptive systems account for all major accessory visual functions in mice. *Nature* 424 76–81. 12808468 10.1038/nature01761 PMC2885907

[B14] Hilgen G. Sorbaro M. Pirmoradian S. Muthmann J. O. Kepiro I. E. Ullo S. (2017). Unsupervised spike sorting for large-scale, high-density multielectrode arrays. *Cell Rep.* 18 2521–2532. 28273464 10.1016/j.celrep.2017.02.038

[B15] Ichinose T. Hellmer C. B. (2016). Differential signalling and glutamate receptor compositions in the OFF bipolar cell types in the mouse retina. *J. Physiol.* 594 883–894. 26553530 10.1113/JP271458 PMC4753269

[B16] Ichinose T. Fyk-Kolodziej B. Cohn J. (2014). Roles of ON cone bipolar cell subtypes in temporal coding in the mouse retina. *J. Neurosci.* 34 8761–8771. 24966376 10.1523/JNEUROSCI.3965-13.2014 PMC4069354

[B17] Jain V. Srivastava I. Palchaudhuri S. Goel M. Sinha-Mahapatra S. K. Dhingra N. K. (2016). Classical photoreceptors are primarily responsible for the pupillary light reflex in mouse. *PLoS One* 11:e0157226. 10.1371/journal.pone.0157226 27295136 PMC4905644

[B18] Kiuchi K. Yoshizawa K. Shikata N. Matsumura M. Tsubura A. (2002). Nicotinamide prevents N-methyl-N-nitrosourea-induced photoreceptor cell apoptosis in Sprague-Dawley rats and C57BL mice. *Exp. Eye Res.* 74 383–392. 12014919 10.1006/exer.2001.1127

[B19] Kostic C. Crippa S. V. Martin C. Kardon R. H. Biel M. Arsenijevic Y. (2016). Determination of Rod and cone influence to the early and late dynamic of the pupillary light response. *Invest. Ophthalmol. Vis. Sci.* 57 2501–2508. 27152964 10.1167/iovs.16-19150 PMC4868103

[B20] La Morgia C. Carelli V. Carbonelli M. (2018). Melanopsin retinal ganglion cells and pupil: clinical implications for neuro-ophthalmology. *Front. Neurol.* 9:1047. 10.3389/fneur.2018.01047 30581410 PMC6292931

[B21] Lucas R. J. Douglas R. H. Foster R. G. (2001). Characterization of an ocular photopigment capable of driving pupillary constriction in mice. *Nat. Neurosci.* 4 621–626. 11369943 10.1038/88443

[B22] Lucas R. J. Hattar S. Takao M. Berson D. M. Foster R. G. Yau K. W. (2003). Diminished pupillary light reflex at high irradiances in melanopsin-knockout mice. *Science* 299 245–247. 12522249 10.1126/science.1077293

[B23] Meira L. B. Moroski-Erkul C. A. Green S. L. Calvo J. A. Bronson R. T. Shah D. (2009). Aag-initiated base excision repair drives alkylation-induced retinal degeneration in mice. *Proc. Natl. Acad. Sci. U.S.A.* 106 888–893. 19139400 10.1073/pnas.0807030106 PMC2621254

[B24] Mure L. S. Vinberg F. Hanneken A. Panda S. (2019). Functional diversity of human intrinsically photosensitive retinal ganglion cells. *Science* 366 1251–1255. 31806815 10.1126/science.aaz0898 PMC7120228

[B25] Pang J. J. Gao F. Wu S. M. (2004). Light-evoked current responses in rod bipolar cells, cone depolarizing bipolar cells and AII amacrine cells in dark-adapted mouse retina. *J. Physiol.* 558 897–912. 15181169 10.1113/jphysiol.2003.059543 PMC1665016

[B26] Pinheiro H. M. Da Costa R. M. (2021). Pupillary light reflex as a diagnostic aid from computational viewpoint: a systematic literature review. *J. Biomed. Inform.* 117:103757. 10.1016/j.jbi.2021.103757 33826949

[B27] Prigge C. L. Yeh P. T. Liou N. F. Lee C. C. You S. F. Liu L. L. (2016). M1 ipRGCs influence visual function through retrograde signaling in the retina. *J. Neurosci.* 36 7184–7197. 27383593 10.1523/JNEUROSCI.3500-15.2016 PMC4938862

[B28] Qiu X. Kumbalasiri T. Carlson S. M. Wong K. Y. Krishna V. Provencio I. (2005). Induction of photosensitivity by heterologous expression of melanopsin. *Nature* 433 745–749. 15674243 10.1038/nature03345

[B29] Smith S. B. Yielding K. L. (1986). Retinal degeneration in the mouse. A model induced transplacentally by methylnitrosourea. *Exp. Eye Res.* 43 791–801. 3803463 10.1016/s0014-4835(86)80010-0

[B30] Smith S. B. Hashimi W. Yielding K. L. (1988). Retinal degeneration in the mouse induced transplacentally by N-methyl-N-nitrosourea: effects of constant illumination or total darkness. *Exp. Eye Res.* 47 347–359. 3181324 10.1016/0014-4835(88)90047-4

[B31] Tao Y. Chen T. Fang W. Peng G. Wang L. Qin L. (2015). The temporal topography of the N-Methyl- N-nitrosourea induced photoreceptor degeneration in mouse retina. *Sci. Rep.* 5:18612. 10.1038/srep18612 PMC4685653 26685797

[B32] Uehara N. Miki K. Tsukamoto R. Matsuoka Y. Tsubura A. (2006). Nicotinamide blocks N-methyl-N-nitrosourea-induced photoreceptor cell apoptosis in rats through poly (ADP-ribose) polymerase activity and Jun N-terminal kinase/activator protein-1 pathway inhibition. *Exp. Eye Res.* 82 488–495. 16168987 10.1016/j.exer.2005.08.006

[B33] Wang F. Li E. De L. Wu Q. Zhang Y. (2021). OFF-transient alpha RGCs mediate looming triggered innate defensive response. *Curr. Biol.* 31 2263–2273.e3. 33798432 10.1016/j.cub.2021.03.025

[B34] Zhu Y. Tu D. C. Denner D. Shane T. Fitzgerald C. M. Van Gelder R. N. (2007). Melanopsin-dependent persistence and photopotentiation of murine pupillary light responses. *Invest. Ophthalmol. Vis. Sci.* 48 1268–1275. 17325172 10.1167/iovs.06-0925

